# Cognitive, physical and disability trajectories in community-dwelling elderly people

**DOI:** 10.1007/s40520-021-01804-3

**Published:** 2021-02-16

**Authors:** Ottavia Eleonora Ferraro, Antonio Guaita, Simona Villani

**Affiliations:** 1 Unit of Biostatistics and Clinical Epidemiology, Department of Public Health, Experimental and Forensic Medicine, University of Pavia, via Forlanini 2, 27100 Pavia, Italy; 2 Golgi Cenci Foundation, Abbiategrasso (Milan), Italy

**Keywords:** Multi-trajectories, Group-based trajectory models, Elders, Dementia

## Abstract

**Backgrounds and aims:**

Health trajectories in aging, rather than single time-point assessments, could be early indicators of the onset of conditions such as dementia. The aim of this study was to identify different aging trajectories and to investigate their influence on the cumulative incidence of dementia.

**Methods:**

We evaluated data referring to 993 elders from the InveCe.Ab study cohort. All subjects were free from dementia at baseline and re-assessed on at least one other occasion thereafter. Cognitive function was assessed using the Mini-Mental State Examination (MMSE), physical function using the Walking Speed Test (WST), and disability on the basis of the Activities of Daily Living (ADL) score. To describe the different courses of the three outcomes combined, the Group-Based Trajectory Model (GBTM) method was applied. We looked for differences in age, gender, education, ApoE-e4 carrier status and obesity, and then investigated the influence of the observed trajectories on the incidence of dementia.

**Results:**

Three trajectories were identified: a “good” scenario was observed in 703 (70.2%) individuals, who showed substantially stable cognitive and physical function and no disability; an “intermediate” scenario in 248 subjects (25.5%), who recorded a longer walking time, lower MMSE score, and a one-point higher ADL score; and a “severe” scenario in 42 elders (4.3%), who recorded declines in all the outcomes. Female gender, obesity and low education were most represented in the “severe” group. ApoE-e4 carrier status showed no difference between groups. The estimated cumulative incidence of dementia was higher in the “severe” (37%) than in the “intermediate” (7%) and “good” (< 1%) scenarios.

**Conclusions:**

Using simple measurements, we built different aging trajectories, and observed that the worst performers had the highest incidence of dementia. Better knowledge of trajectories of aging would be useful for preventive interventions aimed at promoting healthier aging.

## Introduction

Worldwide, the older segment of the adult population is rapidly expanding in number, proportion or both [1]. The old-age population is increasing at a considerably faster rate than the world’s total population, and is expected to increase twofold in the next 30 years [2].

Although cohort comparisons suggest that the debilitating effects of senescence are now increasingly delayed to later ages [3], and a “compression of morbidity” theory highlights a progressive rise in the average age at onset of disabling and chronic diseases [4], in reality, the extra years people are now living do not necessarily bring them good physical health, good cognitive functioning and/or psychosocial well-being. Indeed, older adulthood continues to be associated with functional decline and health problems. In particular, the positive overall scenario of greater longevity is marred by the increasing incidence of dementia [5].

As highlighted by a 2010 document published by the United Nations, fostering physiological and psychosocial well-being across the entire lifespan may help to mitigate the effects of this global demographic shift, and is therefore crucial from a public health, public policy and economic perspective [2]. It is very clear that the quality of aging, understood as a global process, diverges greatly from the quality of aging evaluated on the basis of single average parameters, and major challenges remain in understanding the reason for this. Some studies have identified aging trajectories in specific functional areas, such as cognition [6], or investigated the influence, on them, of specific factors such as sex [7] or education [6]. The prevalence of dementia among individuals aged ≥ 60 years has been found to vary within a narrow range (5–7%) in most world regions. It has been estimated that 35.6 million people worldwide were living with dementia in 2010, with this number expected to almost double every 20 years, to 65.7 million in 2030 and 115.4 million in 2050 [8]. Dementia will thus continue to have a considerable social and economic impact [9].

Against this background, the relationship between aging and dementia is obviously of interest, and physical and cognitive aging trajectories [9], as well as diseases and trajectories of disability [10], have been studied as potential predictors of dementia. However, to our knowledge, no studies have identified or evaluated trajectories of aging from a multidimensional (physical, cognitive and functional) perspective, seeking to ascertain their possible relationship with the incidence of dementia.

The aim of this study was to identify and characterize different trajectories of aging by applying a multi-trajectory statistical model to data drawn from a multidimensional longitudinal study. A secondary aim was to investigate the influence of the different trajectories on the incidence of dementia.

## Methods

### Population and study design

Data for this study were drawn from the population-based multidimensional cohort study InveCe.Ab (Invecchiamento Cerebrale in Abbiategrasso, i.e., Brain aging in Abbiategrasso, Clinical Trial.gov NCT01345110), conducted by the Golgi-Cenci Foundation. The study protocol is detailed elsewhere [11]. Briefly, the population enrolled at baseline (1 November 2009 to the end of 2010) comprised 1321 older adults born between 1935 and 1939, and living in Abbiategrasso, a town of 33,000 inhabitants situated near Milan, in northern Italy. The subjects were invited to attend two follow-up evaluations, in 2012 and 2014, respectively.

Of the original 1321 patients, 993 met the criteria for inclusion in the present study, having attended at least one follow-up evaluation, and been free from dementia at baseline.

All the participants gave their informed consent. The “InveCe.Ab” study protocol was approved by the Ethics Committee of the University of Pavia. The study procedures were in accordance with the principles outlined in the Declaration of Helsinki of 1964 and its subsequent amendments.

### Data collection

Social, bio-clinical and neuropsychological data about the participants were collected by specially trained social interviewers, geriatricians and psychologists at each phase of the study. The evaluations, each lasting three and a half hours, divided into two sessions, took place at the Golgi-Cenci Foundation in Abbiategrasso. In just a few cases, evaluations were carried out at home. All non-instrumental information was collected by means of a bespoke questionnaire.

### Endpoints variables

The primary endpoint was to build aging trajectories by combining cognitive functioning, physical functioning and disability trajectories. Cognition was assessed using the Mini-Mental State Examination (MMSE) [12]. A proxy for physical function was derived from the Walking Speed Test (WST): we considered the time, in seconds, taken to walk back and forth along a special five-meter pathway without pausing. This was part of a talking-while-walking test [13]. The Activities of Daily Living (ADL) score [14] was taken as a measure of disability.

The socio-demographic factors considered in this research were: gender (female vs male), age in years at baseline, and years of education. These data were collected from the social questionnaire or municipal registry, as appropriate.

Obesity corresponded to a Body Mass Index (BMI) of ≥ 30 kg/m^2^ [15]. Presence vs absence of the ApoE-e4 allele was ascertained through real-time PCR (Applied Biosystems) analysis of DNA extracted from each patient’s blood sample.

### Statistical analysis

The trajectories of the selected participants were implemented using the group-based trajectory model (GBTM) method [16]. Since three outcomes were studied together, the multi-trajectory model [17] was used; this assumes a non-parametric maximum likelihood estimator to design the distribution of group trajectories, using a finite mixture model of unknown order *J (number of possible groups)*.

The outcomes were conditionally independent at the level of the latent trajectory group, but not at population level, due to a latent construct of trajectory group membership.

The MMSE score (cognitive function) and the WST time in seconds were modeled through a censored normal distribution, and the ADL score as a zero-inflated Poisson distribution. For all the models fitted, dementia cumulative incidence was introduced as a cross-sectional outcome [18].

The Bayesian information criterion (BIC) was the conventional index used to select the best model: the highest values of this index should provide the best reasonable number of groups [19, 20].

The ApoE-e4, obesity and demographic variables were analyzed to profile the groups identified by the trajectory model.

The Relative Rate Ratio (RRR) with 95% Confidence Interval (95%CI) was reported for the multinomial logistic model in which the “best-performance” group was used as reference.

Analyses were performed using Stata^®^, version 15 (StataCorp LP, College Station, TX). The Stata plugin used to estimate the GBTM was named “TRAJ”.

## Results

### Characteristics of the participants

Table 1 shows the main socio-demographic characteristics of the InveCe.Ab cohort members enrolled in the present study.

**Table 1:** Characteristics of the InvCe.Ab cohort members free from dementia at baseline and with data from at least one follow-up assessment

Variables	Baseline 2010	First follow-up 2012	Second follow-up 2014
MMSE score^a^	*n* = 954	*n* = 966	*n* = 897
Median (25th–75th)	29 (27–29)	29 (27–29)	28 (26–29)
Walking Speed Test (s)	*n* = 985	*n* = 965	*n* = 863
Median (25th–75th)	15.00 (13.00–18.00)	15.00 (13.03–17.72)	15.34 (13.36–18.50)
Disability in ADL score^b^	*n* = 993	*n* = 972	*n* = 901
Median (25th–75th)	0 (0–0)	0 (0–0)	0 (0–1)
Obesity	*n* = 993	*n* = 970	*n* = 877
Yes % (*n*)	15.91% (158)	15.98% (155)	15.73% (138)

^a^MMSE range score (0–30)

^b^ADL range score (0–6)

### Multi-trajectories

The final multi-trajectory model was based on 993 subjects with data from at least two assessments including the baseline one (BIC = -14025.82). Three group trajectories, of different orders, were identified on the basis of the trends of the three outcomes, as reported in Table 2.

**Table 2:** Trajectory shapes of the three outcomes in the three groups identified

Model	Trajectory shapes^a^		
	First order Good scenario *n* = 703	Second order Intermediate scenario *n* = 248	Third Order Severe scenario *n* = 42
MMSE score	1	1	1
Walking Speed Test	0	0	1
ADL Score	1	1	1

^a^Possible trajectory shapes; 0 = zero-order; 1 = linear

The multi-trajectory analysis showed a linear trajectory of MMSE and ADL in all groups, whereas the Walking Speed Test showed a linear trajectory only in the “severe” scenario group.

The “good” scenario group displayed the best trend in all outcomes over the follow-up period, showing no disability. The subjects in the “intermediate” scenario group showed substantial stability on the WST, a 2-point reduction in MMSE score, and needed help in one further ADL at the end of the study.

**Fig. 1:**
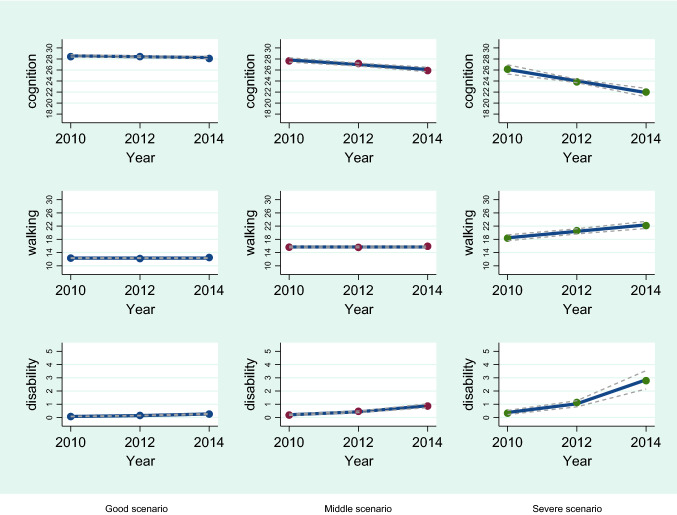
Trends for the single outcomes within (reading down) and between (reading across) the three groups

The worst trajectories over time, for all the outcomes, were identified in the “severe” scenario group, as follows:

- the MMSE score (measuring cognition) was 26 points at baseline and dropped to around 21 points by the end of the study, corresponding to a 1.5-point annual loss;
- walking time increased by around 4 s on average during the entire period;
- disability showed a low level at baseline but was increased by around 3 points at the second follow-up (Fig. 1).

## Group profiles

The “good” scenario group comprised the largest number of elders (*n* = 703): 53.2% were males, and they had a mean age at baseline of 72.7 years (± 1.4 years) and an average 7.6 years of education; 18% (*n* = 129) were ApoE-e4 carriers and almost 12% were obese.

Of the subjects showing an “intermediate” trend (*n* = 253), 65% were females; this group had a mean age at baseline of 72.9 years (± 1.4 years) and 5.8 years (± 2.6 years) of education on average; 24% were obese and 20.16% were ApoE-e4 carriers.

The group displaying a “severe” scenario was much smaller (*n* = 42), largely female (76%), and slightly older than the previous two groups (mean age 73.4 ± 1.2 years). The level of education was low (mean 4.5 ± 3.2 years); 36% were obese and 19% were ApoE-e4 carriers.

The profiles of the subjects belonging to the three groups differed significantly in terms of age, gender, education and the presence of obesity (Table 3).

**Table 3:** Multinomial logistic model for multi-trajectory groups

	RRR	Std. Err	[95% CI]		*P*-value
Intermediate scenario vs good scenario					
Age at baseline	1.11	0.06	0.99	1.23	0.065
Gender (Female)	1.81	0.29	1.32	2.47	< 0.001
ApoE-e4	1.21	0.24	0.83	1.77	0.330
Obesity	2.13	0.42	1.45	3.13	< 0.001
Years of education	0.84	0.03	0.79	0.89	< 0.001
Severe scenario vs good scenario					
Age at baseline	1.43	0.18	1.12	1.82	0.005
Gender (female)	2.93	1.13	1.38	6.24	0.005
ApoE-e4	1.24	0.53	0.54	2.87	0.610
Obesity	3.50	1.27	1.72	7.14	0.001
Years of education	0.63	0.06	0.53	0.75	< 0.001

An older age at baseline carried a 1.4-fold increased risk of showing the worst (“severe”) as opposed to the best (“good”) trajectory (*P* = 0.005; Table 3). Being female carried a 2.9-fold increased risk of being in the “severe” versus the “good” group (*P* = 0.005) and a 1.8-fold increased risk of being in the “intermediate” versus the “good” group (*P* < 0.001). The level of education also differed between the groups: a longer education reduced the risk of being in the “severe” as opposed to the “good” scenario group (*P* < 0.001); similarly, the risk of being in the “intermediate” group rather than the “good” group was inversely related to years of education (*P* < 0.001).

The presence of obesity differed greatly across the three groups: being obese carried a 3.5- and 2.1-fold increased risk of being in the “severe” and “intermediate” group, respectively, versus the “good” group (*P* < 0.001 for both RRR) (Table 3).

### Dementia

Thirty-six subjects had a diagnosis of dementia at the end of the study: 16 (0.8%) of those with a “good” scenario, 14 (6.3%) of those with an “intermediate” scenario, and 6 members (35.3%) of the “severe” scenario group (Table 4).

**Table 4:** Comparison of estimated and observed proportion of people with dementia at the end of the study (*n* = 993)

Group	*n*	Estimated cumulative incidence of dementia using posterior probability from multi-trajectory model (%)	Observed cumulative incidence of dementia (%)
Good scenario	703	0.7 (0.3–1.6)	0.8
Intermediate scenario	248	7.3 (4.7–11.2)	6.3
Severe scenario	42	37.2 (24.3–52.1)	35.3

According to the fitted multi-trajectory model, the three trajectory groups showed specific relationships with the cross-sectional outcome of dementia at the end of the second follow-up [16, 17]. In each of the three scenario groups, the estimated proportion of people with dementia was found to overlap the observed occurrence of dementia (Table 4). It was significantly different in the three groups over time, being found to increase with the severity of the elders’ conditions.

## Discussion

The main findings of this study can be summarized in the following points:

1. Three distinct trajectories of aging were identified: “good”, “intermediate”, “severe”. Most of the participants showed the “good” trajectory.
2. Female gender, obesity and lower education were more represented in the “intermediate” and “severe” trajectories.
3. ApoE-e4 allele carrier status was not associated with any of the three trajectories.
4. Dementia was more represented in the “severe” trajectory group.

The present study showed that most of the Italian elders included in the InveCe.Ab cohort aged in a stable way, preserving good cognitive status, good physical performance, and showing no impairment in ADL. This is consistent with the findings of Christensen et al. [21], who reported that people aged 65–85 years in the last 20 years enjoyed a better quality of life compared with a previous cohort of seniors.

The GBTM statistical approach, starting from cognitive, functional and disability trajectories, clearly identified three scenarios of aging: “good”, with substantially no change over time; “intermediate”, characterized by moderate impairment of cognition and disability; and “severe”, in which there was worsening of all three dimensions. Taken singly, each of the considered dimensions is important in the aging process and has been studied in depth in geriatric research. Trajectories of cognition [6], disability [10] and functional decline [22] together determine the quality of the aging process as a whole, and they are crucial targets of preventive and clinical geriatric medicine. Like ours, all the aforementioned studies, which highlight the reciprocal connection between these dimensions and trajectory-based successful aging, used multidimensional indicators [23].

With regard to the profiles deriving from the multi-trajectory model, the “severe” scenario group had the largest proportion of subjects with dementia (37% versus 7% in the “intermediate” and 1% in the “good” scenarios). The analysis of estimated versus observed rates of dementia showed good agreement, confirming the reliability of the trajectories as predictors of dementia onset, and showing the existence of a relationship between these elderly subjects’ trajectories and their likelihood of developing dementia.

Overall, we demonstrated, within our study population, different trajectories of aging, which have different implications. Obesity and education, known to be important risk/protective factors in the general population, showed different distribution patterns in the three trajectory groups, characterized by increasing rates of obesity and of low education from the “good” to the “severe” scenario. This is in agreement with the findings of other studies evaluating the possible connection between obesity and cognitive and physical decline [24–27]. A higher level of education is a well-known protective factor against cognitive decline [28–30], and a contributing factor to successful aging [31] and longevity [32].

To our knowledge, this is the first attempt to identify trajectories of aging using the GBTM statistical method and starting from the trajectories of three different dimensions of health in the elderly. The variables chosen for this analysis, being very simple and usually present in geriatric evaluations, facilitated the building of the trajectories. Furthermore, the GBTM method allowed us to identify the highest risk of dementia in the group showing the worst scenario.

The value of trajectories as opposed to single-point observations was recently demonstrated in a Canadian study conducted in 154 community-dwelling older people followed up for five years [9]. The findings of that study showed that the incidence of dementia could be reliably derived from cognitive and functional trajectory trends observed over time, whereas a “single time-point assessment was not sufficient to detect individuals at high risk of dementia”.

Furthermore, compared with a single time-point data evaluation, which can only detect associations, the GBTM method, being able to trace, over time, shared or different clinical characteristics between patients belonging to different groups, might find useful clinical application. Indeed, the type of longitudinal analysis we carried out may serve to clarify the factors on which to focus to identify, and subsequently promote, the best aging trajectory.

Our study has several limitations, the first being the presence of missing data in the follow-up assessments. To evaluate the possible effect of attrition bias typical of studies involving elders, the study participants were compared with the rest of the InveCe.Ab cohort (not enrolled in the present study), and no difference was found [11]. Information bias was controlled in this study by means of dual diagnostic assessment (i.e., by a psychologist and geriatrician) and, when necessary, by contacting family doctors. Finally, selection bias was avoided using a careful recruitment method involving direct contact with the subjects, which resulted in a high response rate, around 80% [33].

Second, differences emerged in the numbers of subjects displaying the different trajectories, a circumstance that could result in wider confidence intervals and less precision.

Third, the data concern an age-homogeneous population living in a restricted area, which may well reduce the generalisability of the results.

On the other hand, the study has several strengths. First, the evaluation was carried out by specially trained social interviewers, geriatricians and psychologists (the same ones at each of the three assessment times). Second, the baseline recruitment rate was very high—over 80% of the eligible subjects. Third, the data showed good agreement between the different statistical analyses, and the relationship between the trajectory groups and the cumulative incidence of dementia was clearly demonstrated.

## Conclusions

The present investigation of aging trajectories among Italian elderly people is a first attempt to address this topic from a multi-outcome perspective. This approach allowed us to describe different health status profiles, associated with certain socio-demographic characteristics, and their possible influence on dementia incidence.

Data gathered over longer observation periods, possibly through yearly assessments, may offer more accurate results and make it possible to identify the period of time in which changes begin to affect quality of life. Further explorations in larger and different cohorts are needed to improve knowledge of other aspects related to older people’s health and aging and to confirm the usefulness of this methodology.
